# Quantifying physical activity needed to mitigate genetic risk for obesity

**DOI:** 10.21203/rs.3.rs-2986582/v1

**Published:** 2023-06-08

**Authors:** lide Han, Jeffrey Annis, Hiral Master, Andrew Hughes, Dan Roden, Paul Harris, Douglas Ruderfer, Evan Brittain

**Affiliations:** VANDERBILT UNIVERSITY MEDICAL CENTER; Vanderbilt University Medical Center; Vanderbilt University Medical Center; Vanderbilt University Medical Center; Department of Biomedical Informatics, Vanderbilt University Medical Center, Nashville, TN; Vanderbilt University Medical Center; Vanderbilt University Medical Center; VANDERBILT UNIVERSITY MEDICAL CENTER

## Abstract

Despite consistent public health recommendations, obesity rates continue to increase. Physical activity (e.g. daily steps) is a well-established modifier of body weight. Genetic background is an important, but typically uncaptured, contributor to obesity risk. Leveraging physical activity, clinical, and genetic data from the All of Us Research Program, we measured the impact of genetic risk of obesity on the level of physical activity needed to reduce incident obesity. For example, we show that an additional 3,310 steps per day (11,910 steps total) would be needed to mitigate a 25% higher than average genetic risk of obesity. We quantify the number of daily steps needed to mitigate obesity risk across the spectrum of genetic risk. This work quantifies the relationship between physical activity and genetic risk showing significant independent effects and provides a first step towards personalized activity recommendations that incorporate genetic information to reduce incident obesity risk.

The World Health Organization declared obesity as the greatest threat to the health of Westernized nations.^1^ In the U.S., obesity accounts for over 400,000 deaths per year and affects nearly 40% of the adult population. Despite the modifiable nature of obesity through diet and exercise, rates have continued to increase.

Greater physical activity reduces incident obesity with prior work suggesting taking around 8,000 steps substantially mitigates this risk.^2,3^ However, substantial variability in obesity risk exists, even among individuals with high rates of physical activity, pointing to other contributing factors. Genetic variation is an important contributor to obesity risk with heritability estimates ranging from 40–70%.^4^ Therefore, the amount of physical activity needed to mitigate obesity risk will vary based on genetic background. This has implications for physical activity recommendations on an individual level. Studies to date have implicated an interaction of genetic risk and physical activity using self-reported data and focused on specific genetic loci.^5^

We used linked activity monitoring and genome sequencing data from the *All of Us* Research Program (AoURP) to investigate this important public health question. Activity monitoring was quantified as daily step counts derived from commercial Fitbit devices. Genetic risk was quantified by using a polygenic risk score (PRS) from a large-scale genome wide association study of body mass index (BMI).^6^ We aimed to quantify the average daily step count needed to overcome genetic risk for increased BMI. These findings represent an initial step toward a personalized exercise prescription that integrates genetic information.

Details on the design and execution of the AoURP have been published previously.^7^ Adults enrolled in the AoURP provided informed consent at clinics or regional medical centers across the United States that comprise the AoURP network. This study used AoURP Controlled Tier Dataset version 6 (C2022Q2R6) with data from participants enrolled between May 2018-January 2022. Physical activity data were provided by consenting participants from their own Fitbit devices including the opportunity to share historical data preceding enrollment in AoURP. Additional Fitbit data details and processing are provided in the **Supplemental Materials**.

The analytic cohort included only individuals with a BMI <>30kg/m^2^ at the time activity monitoring began. The primary outcome was incident obesity, defined as a BMI >30kg/m^2^ documented in the medical record at least 6 months after initiation of activity monitoring. The latter stipulation was imposed to reduce the likelihood that obese status predated the beginning of monitoring but had not yet been clinically documented.

Whole genome sequencing and EHR data existed for 2,649 individuals with valid Fitbit data. Details on quality control and filtering of genome sequencing data are provided in the **Supplemental Materials**.

Differences in clinical characteristics across PRS quartiles were assessed using the Wilcoxon or Kruskal-Wallis test for continuous variables and the Pearson chi-square test for categorical variables on the complete case dataset. Cox proportional hazard models were used to examine the association between daily step count (considered as a time-varying variable), PRS, and the time to event for obesity, adjusting for age, sex at birth, average step counts during the first 6 months of monitoring (i.e. baseline step count), cancer status, coronary artery disease status, systolic blood pressure, alcohol use, education level, and a PRS*mean steps interaction term. Additional analytic details are described in the **Supplemental Materials**.

A CONSORT diagram detailing the creation of the analytic dataset is provided in the Supplementary Materials (**Supplementary Figure 1**). We identified 1,297 participants of European ancestry who were free of obesity at baseline, agreed to link Fitbit and EHR data, and with available genome sequencing. The cohort was mostly female (72%) and middle aged (52 years IQR[35, 63]). Across the entire sample, the BMI based PRS explained 8.04% of the phenotypic variation in obesity (beta=1.43, p=1.61×10^−26^). Clinical characteristics stratified by BMI PRS are shown in Table 1. The median follow up time was 5.11 years IQR[3.13, 6.63]. Baseline BMI increased from 24.1kg/m^2^ IQR[22.0, 26.3] in the lowest PRS quartile to 26.5 kg/m^2^ IQR[24.3,28.9] in the top quartile (p = 3.32×10^−11^). The incidence of obesity over the study period increased from 13% (n = 43) in the lowest PRS quartile to 41% (n = 134) in the highest PRS quartile (p = 8.65×10^−16^). We observed an incremental decrease in average daily steps when moving from the low PRS quartile to the highest PRS quartile (p = 0.028).

We next modeled obesity risk stratified by PRS percentile using a Cox proportional hazards model with the 50^th^ PRS percentile indexed to a hazard ratio for obesity of 1 (Figure 1A). The relationship between PRS with incident obesity was direct (p = 3.79×10^−5^) and linear (chunk test for nonlinearity was nonsignificant) with a lower risk of obesity at lower PRS. The obesity PRS and average daily step count were both independently associated with obesity risk. The 75^th^ percentile BMI PRS demonstrated a 78% increase in obesity risk (HR: 1.78; 95% CI 1.54, 2.06; = 1.03×10^−14^) when compared with the 25^th^ percentile BMI PRS whereas the 75^th^ percentile average step count demonstrated a 40% reduction in obesity risk (HR:0.6; 95% CI 0.48, 0.77; p = 2.92×10^−5^) when compared with the 25^th^ percentile step count. The PRS*mean steps interaction term was not significant (p = 0.055). We found that individuals with a PRS in the 75^th^ percentile would need to walk an average of 3,310 (range 2,000–7,250) more steps per day (11,910 total) than those at the 50^th^ percentile to reduce the hazard for obesity to 1 (Figure 1B). Conversely, those in the 25^th^ percentile PRS could reach a hazard of 1 by walking an average of 2,260 (range 1,470–4,260) fewer steps than those at the 50^th^ percentile PRS. When assuming an average daily step count of 8,600 (cohort median), those in the 75^th^ percentile PRS had a hazard for obesity of 1.33 (95% CI 1.24, 1.43) whereas those at the 25^th^ percentile PRS had an obesity hazard of 0.75 (95% CI 0.69, 0.80). The average daily step count required to achieve a hazard for obesity of 1 across the full PRS spectrum is shown in Figure 1B. The cumulative incidence of obesity at the 25^th^, 50^th^, and 75^th^ PRS percentiles would be 3.0%, 4.0% and 5.3% in year 1, 11.4%, 15.0% and 19.4% in year 3 and 21.4%, 27.7% and 35.0% in year 5 (**Supplementary Figure 2**).

We examined the combined impact of daily step counts and genetic risk for obesity on the incidence of obesity in a large national sample with genome sequencing and long-term activity monitoring data. Lower daily steps and higher PRS were both independently associated with increased risk of obesity with the obesity PRS having a larger effect. As the PRS increased, the number of daily steps needed to prevent obesity increased. By combining these data sources, we were able to derive an estimate of the daily step count needed to reduce the risk of obesity based on an individual’s genetic background.

These results suggest that population-based recommendations that do not account for genetic background may not accurately represent the amount of physical activity needed to reduce the risk of obesity. For example, we show that at an assumed daily step count of 8,600, the hazard of obesity varies by nearly 60% between the 25^th^ and 75^th^ PRS percentiles (HRs 1.33 vs. 0.75, respectively) and that the absolute number of steps needed to prevent obesity varies by ~6,000 daily steps (Figure 1B) across the full spectrum of genetic risk. Population-based exercise recommendations may over- or underestimate physical activity needs depending on one’s genetic background. Integration of activity and genetic data could facilitate personalized activity prescriptions that account for an individual’s genetic profile. The widespread and growing use of wearable devices and the incorporation of genetic information into the EHR may soon facilitate testing the value of personalized activity prescriptions. Moreover, the combined impact of physical activity and genetics on risk of other cardiovascular risk factors (e.g. hypertension and diabetes) warrants study to further inform personalized recommendations.

The most important limitation of this work is the lack of diversity in our cohort, which reflects the demographics of individuals who owned a Fitbit at the time of enrollment. These findings will need validation in a population that includes participants who are historically underrepresented in biomedical research. Because this study was observational and retrospective, we cannot account for unmeasured confounding and the potential for reverse causation still exists. We attempted to diminish the latter concern by excluding those with prevalent obesity and incident cases that occurred within the first 6 months of monitoring. Genetic risk was simplified to be specific to the outcome of obesity, however, genetic risk for other cardiometabolic conditions could also inform obesity risk and these are not currently accounted for. Finally, many other non-genetic factors including diet, sleep, and socioeconomic status (among others) that contribute to obesity risk were not included, reducing the complete explanatory power of the model and opportunity for other potential interventions.

In summary, these findings support the notion that higher daily step counts can mitigate genetic risk for obesity. These results have important clinical and public health implications and may offer a novel strategy for addressing the obesity epidemic by informing activity recommendations that incorporate genetic information. Lastly, we provide a foundation for future studies to validate these findings in a genetically, sociodemographically, and clinically diverse population.

## Figures and Tables

**Figure 1:**
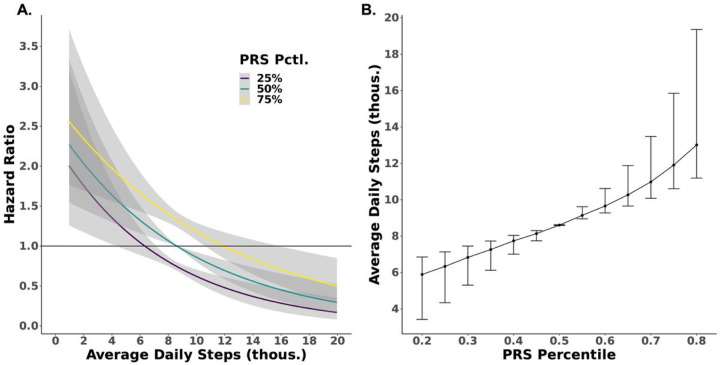
**Risk of Incident Obesity Modeled by Average Daily Step Count and Polygenic Risk Scores** 1A) Hazard for obesity modeled according to average daily step counts and 25^th^, 50^th^, and 75^th^ percentile polygenic risk score (PRS) for body mass index. Shaded regions are 95% confidence intervals. Model is adjusted for age, sex, average baseline step counts, cancer status, coronary artery disease status, systolic blood pressure, alcohol use, education level, and a PRS*mean steps interaction term. 1B) Average daily step count and PRS indexed to a hazard for obesity of 1. Error bars represent 95% confidence intervals.

**Table 1:** Participant Characteristics Across PRS Quartiles

	PRS Quartile 1	PRS Quartile 2	PRS Quartile 3	PRS Quartile 4	P Value
**Participants (*N)***	325	324	324	324	
**Age**	54.5 (39.1 – 62.8)	53.0 (36.8 – 63.0)	50.6 (34.9 – 61.7)	50.1 (33.6 – 62.0)	0.09
**Race**					0.74
**Other, n**	<20*	<20	<20	<20	
**White, n**	310–330	310–330	310–330	310–330	
**Sex, n (%) female**	223 (69)	242 (75)	244 (75)	230 (71)	0.18
**BMI, kg/m2**	24.1 (22.0 – 26.2)	25.0 (22.9 – 27.4)	25.2 (23.2 – 28.3)	26.5 (24.3 – 28.9)	3.32×10^−11^
**Education**					0.10
**College Degree, n(%)**	269 (83)	255 (79)	264 (82)	239 (74)	
**Some College, n**	40–60	50–70	40–60	60–80	
**No College, n**	<20	<20	<20	<20	
**Baseline conditions**					
**Coronary Artery Disease, n**	< 20	< 20	< 20	< 20	0.43
**Cancer, n (%)**	80 (25)	88 (27)	73 (23)	57 (18)	0.03
**Smoking > 100 cigarettes, n(%)**	85 (27)	88 (27)	99 (31)	95 (30)	0.60
**Alcohol ^3^1 drink, n(%)**	319 (98)	315 (98)	318 (98)	311 (97)	0.78
**Fitbit Monitoring Duration (years)**	5.1 (3.0 – 6.6)	4.8 (3.0 – 6.6)	5.1 (3.4 – 6.5)	5.4 (3.5 – 6.8)	0.25
**Average Daily Steps**	8565 (6790 – 10581)	8435 (6549 – 10262)	8136 (6310 – 10114)	8018 (6345 – 10129)	0.12

Data are median (IQR) for continuous variables and frequency (percentage) for categorical variables. Statistical analyses used include: Kruskal-Wallis test and Pearson test. Abbreviations: body mass index (BMI) and polygenic risk score (PRS).

* We report <20 or values in range because *All of Us* Data and Statistics Dissemination Policy does not allow displaying exact participant counts <20 or participant count that would allow another count value to be derived revealing a count less than 20 to protect participants’ privacy.

## References

[1] Obesity: preventing and managing the global epidemic. Report of a WHO consultation. i-xii-1–253 (2000). 11234459

[2] Master H. Association of step counts over time with the risk of chronic disease in the All of Us Research Program. Nat Med 28, 2301–2308 (2022). 36216933 10.1038/s41591-022-02012-w PMC9671804

[3] Cleven L., Krell-Roesch J., Nigg C. R. & Woll A. The association between physical activity with incident obesity, coronary heart disease, diabetes and hypertension in adults: a systematic review of longitudinal studies published after 2012. Bmc Public Health 20, 726 (2020). 32429951 10.1186/s12889-020-08715-4 PMC7238737

[4] Loos R. J. F. & Yeo G. S. H. The genetics of obesity: from discovery to biology. Nat Rev Genet 23, 120–133 (2022). 34556834 10.1038/s41576-021-00414-z PMC8459824

[5] Ahmad S. Gene × Physical Activity Interactions in Obesity: Combined Analysis of 111,421 Individuals of European Ancestry. Plos Genet 9, e1003607 (2013). 23935507 10.1371/journal.pgen.1003607 PMC3723486

[6] Yengo L. Meta-analysis of genome-wide association studies for height and body mass index in ~700000 individuals of European ancestry. Hum Mol Genet 27, 3641–3649 (2018). 30124842 10.1093/hmg/ddy271 PMC6488973

[7] Investigators A. of U. R. P. The “All of Us” Research Program. New Engl J Med 381, 668–676 (2019). 31412182 10.1056/NEJMsr1809937 PMC8291101

